# Knowledge and Attitudes Regarding Childhood Hearing Loss Among Parents in Western Saudi Arabia

**DOI:** 10.7759/cureus.91644

**Published:** 2025-09-05

**Authors:** Manar Alghamdi, Sarah S Almohammdi, Noor S Alharbi, Fatimah Klantan, Zubida Binsiddiq, Mahmoud Alreefi, Malak J Gazzaz, Naif A Bawazeer

**Affiliations:** 1 Medicine and Surgery, Umm Al-Qura University, Makkah, SAU; 2 Otolaryngology - Head and Neck Surgery, King Abdulaziz University, Medical College, Rabigh, SAU; 3 Otolaryngology - Head and Neck Surgery, Ophthalmology, Umm Al-Qura University, Makkah, SAU

**Keywords:** attitude, childhood hearing loss, knowledge, parents, saudi arabia

## Abstract

Childhood hearing loss (HL) can severely hinder social, cognitive, and communication skills. Parental awareness and knowledge have a significant impact on the results and repercussions. The purpose of this study is to assess parents' attitudes and knowledge about childhood HL in Western Saudi Arabia.

The questionnaire was adapted from a previously validated survey. It was translated into Arabic and culturally adapted to the Saudi context after expert review. The tool consisted of three sections: informed consent, demographic information, and assessment of parents’ knowledge and attitudes toward childhood HL, and it was distributed over social media between July and August of 2024. IBM SPSS Statistics for Windows, Version 29 (Released 2022; IBM Corp., Armonk, New York, United States) was used to analyze the data.

Our study of 522 participants revealed that parents in Western Saudi Arabia have good knowledge and positive attitudes regarding childhood HL. Most parents supported the use of hearing aids (96%, n=501) and cochlear implants (92.7%, n=484). However, 20% of parents expressed negative attitudes toward newborn hearing screening tests. Socioeconomic factors significantly influenced awareness levels, with higher family income predicting more positive attitudes toward hearing aids (B=0.623, p=0.045). Age also impacted both knowledge (p=0.004) and attitudes (p=0.020), with younger parents demonstrating greater awareness and more positive perspectives. The primary sources of information were doctors/health centers (28%) and social media applications (27%).

Parents in Western Saudi Arabia showed a high level of awareness and favorable attitudes about childhood HL. To fill in the identified information gaps about childhood HL, a few educational initiatives are still required.

## Introduction

Due to delays in speech, language, and cognitive development, children with hearing loss (HL) experience a considerable reduction in their quality of life. In this study, childhood refers to the period from birth up to puberty, which is the critical window for speech, language, and cognitive development, also referred to as an invisible disability [1]. If untreated, HL can cause a significant impairment by affecting a child's academic performance, emotional and social development, and job chances [2]. The World Health Organization reports that with a prevalence of 1.7 cases per 1,000 newborns, there were 466 million HL cases globally in 2017, including 34 million children [3,4]. According to earlier studies conducted in Saudi Arabia, between 7.7% and 13% of children have sensorineural HL [5,6].

These long-term impairments can be lessened by early identification and treatment of HL through newborn hearing screening programs (NHSPs). NHSPs have been required at all Saudi Arabian hospitals providing obstetric services since 2016. Furthermore, the improvement of outcomes for children with HL depends on early hearing rehabilitation using assistive technologies such as cochlear implants and hearing aids [3,4]. Parental knowledge has a major impact on the effectiveness of universal newborn hearing screening (UNHS) and hearing rehabilitation programs. Lack of awareness about these approaches increases the likelihood that parents may have unfavorable opinions, which could delay diagnosis and treatment and result in worse outcomes for their kids [7,8].

Parental attitudes on childhood HL have been the subject of numerous studies [7-10]. Research conducted in the United Arab Emirates, for instance, revealed varying parental attitudes and awareness regarding pediatric audiology services and childhood HL. It showed that while 65.8% of parents were unaware of the various facets of HL, 34.2% of parents knew enough about it [9]. Additionally, research from around the globe has shown that parents' understanding of the causes of childhood HL varies [9,10]. According to a Solomon Islands study [10], the majority of parents associated childhood HL with otitis media (38%) and family history (64.7%), as well as noise exposure (88.7%). However, there was a marked lack of knowledge of biological issues, such as delayed cry at birth (13.3%) and jaundice (15.3%).

In Saudi Arabia, few studies have investigated parental knowledge and attitudes toward childhood HL, particularly in the western region. Five hospitals in the Al Qassim region participated in a single cross-sectional survey. The study found that although 92.2% of participants expressed favorable opinions about audiology services, 57.6% were not well-informed about childhood HL [1]. The study concluded that a significant information gap still exists, despite parents' typically supportive attitudes toward hearing therapies.

Research highlights the need for parents to be more knowledgeable about childhood HL and to encourage early detection and treatment to enhance outcomes [7-10]. Research on parental awareness and attitudes toward childhood HL in the western part of Saudi Arabia is still lacking, despite this need. To identify obstacles and inform local authorities to lessen the impact of childhood HL, it is imperative to comprehend these gaps. Therefore, the purpose of this study was to assess the knowledge and attitudes of parents in this area of childhood HL.

## Materials and methods

Parents of both sexes were included, but people who were younger than 18 or older than 65, didn't have children, or refused to take part in the study were not. The minimum required sample size was calculated using the OpenEpi version 3.0 calculator (Dean AG, Sullivan KM, Soe MM. OpenEpi: Open Source Epidemiologic Statistics for Public Health, Version, www.openepi.com), based on the estimated population size of Western Saudi Arabia, a 95% confidence interval, and a 5% margin of error. The estimated sample size was 384 participants, which was increased to 400 to allow for potential data loss. Ultimately, data were collected from 522 parents, exceeding the minimum requirement and ensuring robust statistical power. All valid responses (n=522) were included in the final analysis. The study was conducted between July and August 2024. Convenience sampling was applied, with the online questionnaire distributed via social media channels. The sampling frame comprised parents from Western Saudi Arabia, including Mecca, Medina, Jeddah, and Taif. The target group was reached online through social media channels using a self-administered questionnaire in Arabic via a Google Form (Google LLC, Mountain View, California, United States). Three sections made up the questionnaire, which was adapted from a validated study (Appendices 1, 2) [10] and comprised the consent form in the first, demographic information in the second, and an assessment of participants' knowledge and attitudes toward HL in childhood in the third.

The study was approved by the Institutional Research Board of Umm Al-Qura University, Approval No. (HAPO-02-K-012-2024-10-2214). Participants provided informed consent prior to completing the questionnaire. A thorough statistical analysis was performed using both inferential and descriptive methods. Descriptive analysis summarized the participants' demographic details, including gender, age, and other attributes, to provide an overview of the study population. Independent sample t-tests and one-way ANOVA were used to assess differences in mean knowledge and attitude scores across demographic groups, while binary logistic regression was conducted to identify predictors of positive parental attitudes toward hearing aid use. Although a formal normality test was not performed, the large sample size (n=522) justified the use of parametric tests according to the central limit theorem. Statistical significance was defined as a p-value of 0.05 or less, with a 95% confidence interval. Knowledge scores were calculated by assigning one point for each correct answer and zero for incorrect answers. Total scores were categorized using percentiles: high (above the 50th percentile), moderate (25th-50th percentile), and low (below the 25th percentile). This percentile-based categorization was used to provide a relative comparison of participants’ knowledge levels within the sample distribution. Attitude items were scored by assigning one point for each positive response and zero for each negative response. Total scores were then used to classify parental attitudes as either positive or negative. All statistical analyses were conducted using IBM SPSS Statistics for Windows, Version 29 (Released 2022; IBM Corp., Armonk, New York, United States).

## Results

Our study included 522 participants, of whom 411 (78.7%) were females. The mean age was 38.9 ± 10.3 years, 315 (60.3%) had one to three children, and a total of 354 participants (67.8%) reported having a university degree or higher educational qualification. Of them, 190 (36.4%) reported a monthly income of 10,000-20,000 SAR, and 243 (46.6%) were residents of Mecca city (Table 1).

**Table 1:** Sociodemographic and other parameters of participants SAR: Saudi Riyal

Parameters		Frequency (n=522)	Percent
Gender	Female	411	78.7
	Male	111	21.3
Age (Year)	Mean	38.9 (10.3)	
	Range	14-68	
No. of Children	0	21	4.0
	1–3	315	60.3
	> 3	186	35.6
Educational Qualification	Primary to middle school	62	11.9
	High school	106	20.3
	University and above	354	67.8
Monthly Income	<5000 SAR	93	17.8
	5000–10,000 SAR	182	34.9
	10,000–20,000 SAR	190	36.4
	> 20,000 SAR	57	10.9
City of Residence	Mecca	243	46.6
	Medina	149	28.5
	Jeddah	84	16.1
	Taif	46	8.8

Table 2 provides details on parents’ knowledge about childhood HL. The majority, 424 (81.2%) of the participants, were aware that children might be born with or develop HL. However, only 212 (40.6%) were aware of risks associated with viral infections during pregnancy, and just 133 (25.5%) recognized the effects of certain medications. Awareness of jaundice (63, 12.1%) and low birth weight (100, 19.2%) as risk factors was limited. The harmful effects of loud sounds were understood by 430 (82.4%) of the participants, while 326 (62.5%) knew that ear infections could indicate hearing problems. Additionally, 355 (68.0%) linked breastfeeding and preventive measures, yet only 179 (34.3%) linked smoking to ear infections that could lead to HL.

**Table 2:** Assessment of parents’ knowledge about childhood hearing loss

Parent's Knowledge	Correct Response	
	Frequency (n=522)	Percent
Children may be born with a deficiency or loss of hearing immediately at birth or develop hearing loss later.	424	81.2
A child’s extremely high temperature (fever) can cause hearing loss.	374	71.6
Measles or brain infections can cause hearing loss.	317	60.7
A mother infected with some viral infections (such as German measles) during pregnancy can cause hearing loss in her child.	212	40.6
Child taking certain medications or antibiotics can cause hearing loss.	133	25.5
A yellowing skin condition (jaundice) can cause hearing loss.	63	12.1
Infant birth weight of less than 1500 grams may be accompanied by decreased or loss of hearing.	100	19.2
A family history of hearing loss can be a cause of a child’s hearing loss.	394	75.5
Consanguineous marriage can cause hearing loss in the child.	334	64.0
Congenital deformities, including facial deformities, can be a sign of hearing deficiency or loss.	209	40.0
Exposure to loud sounds (noise), especially when listening to music with headphones, can cause hearing loss.	430	82.4
Ear discharge or otitis media can be a sign of decreased or lost hearing.	326	62.5
Repeated infections in a child can cause hearing loss.	339	64.9
Breastfeeding during the first six months can reduce/prevent otitis media and, in turn, reduce the rate of hearing loss.	355	68.0
Smoking around a child can cause a middle ear infection in the child, which may lead to hearing loss.	179	34.3
Routine immunization in childhood can reduce middle ear inflammation and, in turn, reduce the rate of hearing loss.	337	64.6
Speech/language problems can be a sign of hearing deficiency or loss.	397	76.1
Children with hearing impairment or loss can attend public school after receiving treatment.	421	80.7

**Figure 1:**
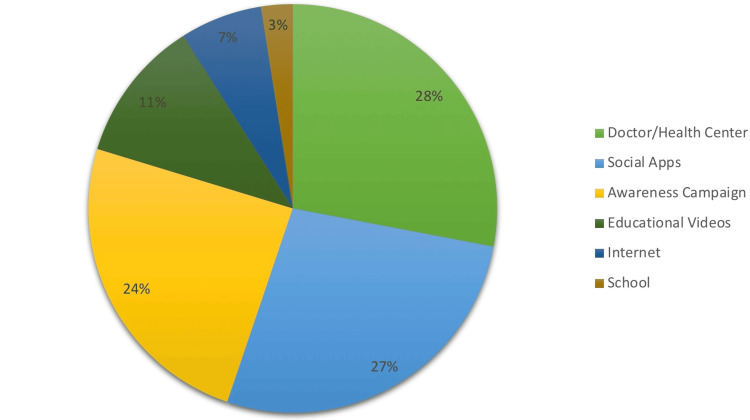
Parents’ source of information about childhood hearing loss

Figure 1 illustrates that doctors or health centers were the primary source of information about childhood HL (146: 28%), followed by social media (140, 27%). Awareness campaigns accounted for 125 (24%), while educational videos accounted for 57 (11%).

**Figure 2:**
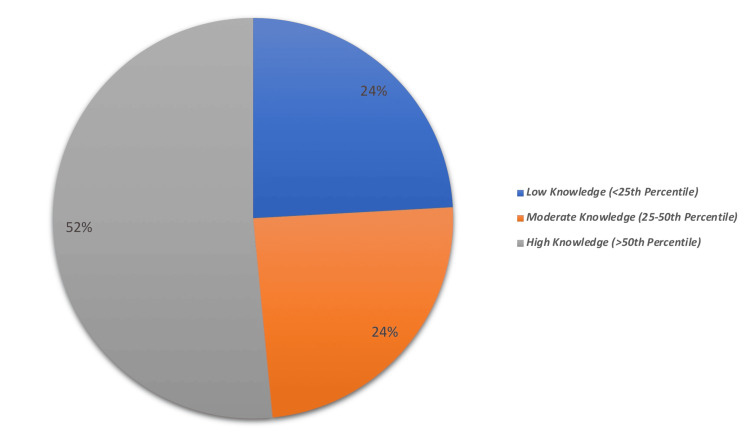
Parents’ overall knowledge level about childhood hearing loss

Figure 2 shows that 271 (52%) of the participants had high knowledge (above the 50th percentile), and 125 (24%) had moderate (25th-50th percentile) or low knowledge (below the 25th percentile) about childhood HL.

**Figure 3:**
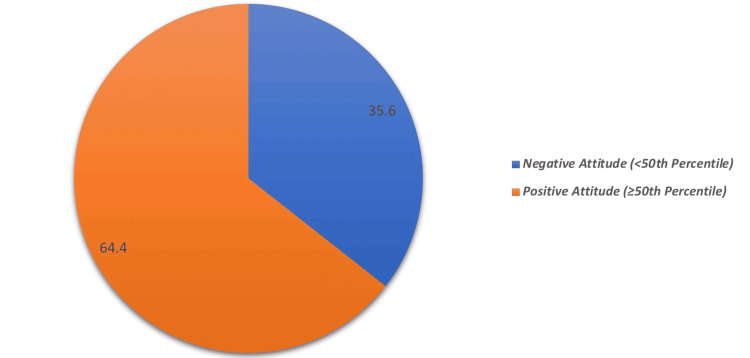
Parents’ overall attitude about childhood hearing loss

Table 3 outlines parents’ attitudes toward childhood HL and related interventions. The majority of the participants, 501 (96.0%), were willing to allow their children to use hearing aids. Similarly, 494 (94.6%) were open to ear surgery, and 484 (92.7%) supported cochlear implants. The importance of early detection was recognized by 489 (93.7%) of parents who had their babies examined after birth, though only 418 (80.1%) supported newborn hearing screening. Figure 3 shows that 336 (64.4%) of parents displayed positive attitudes toward childhood HL, while 35.6% expressed negative attitudes.

**Table 3:** Assessment of parents’ attitudes about childhood hearing loss

Parent's Attitudes		Frequency (n=522)	Percent
Allow hearing aids	Yes	501	96.0
Want more information on hearing loss	Yes	496	95.0
Allow ear surgery	Yes	494	94.6
Baby examined after birth	Yes	489	93.7
Hearing test at school	Yes	484	92.7
Allow cochlear implant	Yes	484	92.7
Newborn hearing screening	Yes	418	80.1

Table 4 demonstrates the association between parents’ knowledge of childhood HL and demographic and socioeconomic factors. It was found that the mean knowledge score was significantly higher among participants with younger age (≤ 30 years), those having one to three children, those with higher monthly income (10,000-20,000 SAR), those from Taif city, and those whose main source of information about children's HL was school.

**Table 4:** Association and difference of knowledge and attitude scores with different features a: independent t-test; b: analysis of variance

Variable		Knowledge Score				Attitude Score			
		Frequency (N)	Mean (SD)	Test	Sig. Value	Frequency (N)	Mean (SD)	Test	Sig. Value
Gender	Female	411	10.07 (4.12)	1.8	0.071a	411	6.45 (0.93)	0.08	0.932a
	Male	111	10.87 (4.39)	-		111	6.44 (1.04)	-	-
Age	Up to 30 years	132	10.63 (4.29)	1.77	0.004b	132	6.50 (1.04)	1.86	0.020b
	31–50 years	316	10.42 (4.15)	-		316	6.49 (0.84)	-	-
	> 50 years	74	8.74 (3.90)	-		74	6.16 (1.19)	-	-
Number of Children	0	21	7.52 (3.71)	5.08	0.006b	21	6.00 (1.70)	5.93	0.003b
	1-3	315	10.48 (4.19)	-		315	6.55 (0.79)	-	-
	>3	186	10.13 (4.14)	-		186	6.32 (1.06)	-	-
Education Level	Primary to middle school	62	9.27 (4.39)	2.82	0.060b	62	6.34 (0.92)	0.71	0.490b
	High school	106	9.88 (4.33)	-		106	6.41 (1.16)	-	-
	University and above	354	10.51 (4.09)	-		354	6.48 (0.89)	-	-
Income	< 5000 SAR	93	9.11 (4.26)	3.7	0.01b	93	6.20 (1.22)	2.82	0.038b
	5000–10,000 SAR	182	10.42 (4.15)	-		182	6.45 (0.95)	-	-
	10,000–20,000 SAR	190	10.76 (3.97)	-		190	6.55 (0.85)	-	-
	> 20,000 SAR	57	9.74 (4.59)	-		57	6.51 (0.68)	-	-
City	Mecca	243	9.56 (4.45)	8.52		243	6.44 (0.86)	1.74	0.157b
	Medina	149	10.70 (4.05)	-		149	6.46 (1.07)	-	-
	Jeddah	84	10.02 (3.65)	-		84	6.32 (1.13)	-	-
	Taif	46	12.72 (2.87)			46	6.72 (0.50)	-	-
Source of Information	Doctor/health center	146	9.95 (4.32)	1.02	0.223b	146	6.46 (0.96)	0.09	0.690b
	Social media apps	142	10.25 (3.86)	-		142	6.49 (1.00)	-	-
	Awareness campaign	128	10.23 (4.51)	-		128	6.42 (0.85)	-	-
	Educational videos	59	10.92 (3.13)	-		59	6.47 (0.82)	-	-
	Internet	34	9.41 (4.84)	-		34	6.21 (1.34)	-	-
	School	13	12.38 (4.93)	-		13	6.62 (0.65)	-	-

Finally, Table 5 presents adjusted sociodemographic predictors of parents’ positive attitudes toward hearing aid use for their children. A higher monthly income was a significant predictor (B=0.623, p=0.045). Binary logistic regression analysis demonstrated that higher monthly income was the only significant predictor of parents’ positive attitudes toward hearing aid use (B=0.623, p=0.045; OR=1.86, 95% CI: 1.02-3.42). Other factors, including gender, age, number of children, and education level, were not statistically significant predictors of attitudes toward hearing aid use.

**Table 5:** Adjusted sociodemographic predictors of parents with a positive attitude toward using hearing aids for their child

Predictors	B	Sig.	Exp(B)	95% CI	
				Lower	Upper
Gender (male)	-0.777	0.140	0.460	0.164	1.289
Age	-0.043	0.089	0.958	0.911	1.007
Number of children	0.038	0.778	1.039	0.796	1.357
Higher educational level	0.434	0.165	1.544	0.836	2.851
Higher monthly income of the family	0.623	0.045	1.864	1.015	3.423

## Discussion

Speech, academic performance, future employment, and social skills are all significantly impacted by HL in children [11]. Even mild or minimal HL (pure-tone average threshold between 15 and 30 dB) is associated with speech and social skills issues [12]. However, 60% of pediatric HL cases are caused by preventable causes that occur before, during, or after pregnancy [13]. Preventing these preventable causes and working toward early detection and intervention are necessary to lessen the long-term impact of childhood HL. Family attitudes and expertise are crucial to support these treatments and ensure that a management plan is followed [14].

Parental attitudes and understanding about HL and its treatment vary globally, according to studies [9,10], and there is a strong correlation between awareness levels and HL outcomes. Nevertheless, to our knowledge, there has never been a global assessment of Saudi Arabian parents' knowledge of childhood HL, especially in the country's west. Our study evaluated parents' attitudes and knowledge regarding HL in children.

With knowledge ratings above the 50th percentile, 52% of parents demonstrated a good level of understanding of childhood HL. Conversely, Alsudays et al. [1] found that 57.6% of parents in the Al Qassim region lacked sufficient knowledge of childhood HL. Similarly, Ayas et al. [9] discovered that 65.8% of the parents in their research had very little knowledge. Local educational initiatives and disparities in the parents' educational and socioeconomic backgrounds may be the cause of these differences. Around 81.2% of parents in our study agreed that children could be born with or develop HL, which is consistent with data from previous studies [1,15], where approximately 63% of parents were aware of congenital causes of HL.

Knowledge of the consequences of high fever and infections, such as measles, is in line with the findings of Joubert et al. [16], who highlighted a high awareness of infectious causes of HL. However, mothers' knowledge of children's HL risk factors, such as measles, was low (22.6%), according to Wang et al. [17].

The current study found that only 12.1% and 25.5% of parents, respectively, recognized jaundice and certain medications as potential causes of HL [18]. However, 78.4% of parents were aware of the ototoxic consequences of several drugs, according to Govender et al. [19]. This emphasizes the need for specialized training programs on the risks related to medications in Western Saudi Arabia. In Al Qassim, 48.6% of mothers and 38.1% of dads cited medication as a potential cause of HL [1]. The regional and cultural differences in Saudi Arabia may be the cause of this disparity in results.

The vast majority of survey respondents had positive opinions regarding both surgery and hearing aids, with 96% of parents expressing openness to them. Similarly, Kaspar et al. [10] found that 94% of parents supported the use of hearing aids, whereas 64% of parents were open to ear surgery to improve hearing. However, the high acceptability (94.6%) of surgical treatments observed in this study may overstate reality because participants were not asked to make informed decisions about potential surgery and its risks and consequences. More than 90% of parents endorsed early diagnostic techniques, including birth and school hearing screenings, highlighting the significance Saudi parents place on early HL identification to improve children's outcomes.

According to recent assessments of NHSPs in Saudi Arabia, 199,034 neonates were examined in 2021, with a 1.87% referral/fail rate and a 92.6% coverage rate [20]. Alanazi [21] reported an NHSP referral/fail rate of 1.33%, despite the fact that the data were limited to two large hospitals in Riyadh. A 2021 study that examined UNHS programs found that since 2016, one million newborns had received testing, with a 96% coverage rate and a 0.7% referral rate [22]. These programs offer early detection and timely intervention, which is essential for social, linguistic, and speech development [23-26]. The current study found that only 80.1% of parents supported newborn screening, indicating the need for further education about NHSPs and their benefits. This finding aligns with a previous report [27] of 18% follow-up default by parents after referral, which was higher than the international benchmark.

Higher family income was found to be a major predictor of favorable attitudes toward the adoption of hearing aids. This conclusion is supported by Zhao et al. [28], who noted that a higher economic level typically correlates with a better chance of exploring hearing aid solutions for children. A study [29] also emphasized the financial barriers to accessing healthcare services for hearing in lower-income countries, where financial limitations significantly influence healthcare choices. It's noteworthy that while this study found no significant correlation between attitudes and educational attainment, a study conducted in Iran [30] found that higher parental education was linked to improved hearing intervention outcomes. Similarly, there were no appreciable differences in attitudes or knowledge based on gender or educational attainment, according to Alsudays et al. [1].

Doctors, social media, and awareness campaigns were the most frequently cited sources of information for parents on children's HL; this underscores the importance of these channels for disseminating health-related information. Noise-induced HL has become a major issue for young adults and adolescents as a result of the widespread use of portable devices that produce hazardous noise levels. This survey indicates that 20% of parents need to be educated on this matter, highlighting the necessity of targeted awareness campaigns.

Limitations

Among other limitations, our cross-sectional design makes it difficult to track changes over time, and our dependence on self-reported data may lead to response biases. Furthermore, because of its focus on metropolitan regions, the study might not accurately reflect rural populations. Additionally, the high proportion of female participants can distort results linked to gender, emphasizing the need for more equal male-to-female ratios in future research.

Implications and future directions

In order to address the parents' apparent information gaps around childhood HL, this study highlights the need for targeted educational programs. Educational programs should focus on raising awareness of the less well-known risk factors for HL, such as medications, jaundice, low birth weight, and noise exposure, in addition to promoting positive attitudes around newborn hearing screening. More research should be done on a larger scale to cover additional geographic regions, such as rural and underserved communities, in order to increase the findings' generalizability. In order to monitor changes in attitudes and knowledge over time, longitudinal studies are also recommended. To provide a more gender-balanced perspective, male participation should be promoted. The general knowledge and treatment of childhood HL could be greatly enhanced by community-based awareness initiatives and the incorporation of hearing health education into ongoing public health campaigns.

## Conclusions

The generally high levels of parental knowledge and positive attitudes toward HL in childhood therapy that we identified in this study are encouraging because these are crucial components in the successful management of childhood HL. However, differences in knowledge of particular risk factors and gaps in awareness highlight the need for improved parental education and targeted public health strategies, and the significant influence of socioeconomic status underscores the need for tailored health education initiatives. In order to ensure equitable access to healthcare and improve outcomes for all children with HL, future strategies should concentrate on bridging socioeconomic and educational gaps.

## Appendices

Appendix 1

Hearing Loss Questionnaire

Demographics information:

          1- Age:

1.          < 25 years

2.          26 - 35 years

3.          36- 45 years

4.          >46 years

         2- Level of school:

1.          Primary school

2.          Secondary school

3.          Tertiary school

           3-City:

1.          Makkah

2.          Jeddah

3.          Taif

Maternal knowledge and attitudes to childhood hearing loss and hearing services:

1.          Babies can be born with hearing loss                                                     Yes              No             Unsure

2.          High fever can cause hearing loss

3.          Measles can cause hearing loss

4.          Maternal rubella can cause hearing loss

5.          Drugs/medication can cause hearing loss

6.          Jaundice can cause hearing loss

7.          Delayed crying can cause hearing loss

8.          Family history can cause hearing loss

9.          Noise exposure can cause hearing loss

10.        Evil spirits can cause hearing loss

11.        Curses can cause hearing loss

12.        Ear discharge and otitis media can cause hearing loss

13.        Recurrent flu can cause hearing loss

14.        Breastfeeding for the first 6 months can reduce/prevent otitis media

15.        Smoke (tobacco/woodfire) can predispose to otitis media

16.        Routine childhood immunization can reduce otitis media

17.        Hearing loss can be identified soon after birth

18.       Speech/language problems can be a sign of hearing loss

19.       Treatment of hearing loss is available

20.      Children with hearing loss can attend school

21.       I would like my baby tested soon after birth

22.      I would accept the otoacoustic emissions (OAE) hearing screening test for my baby

23.      I would like my child tested at school

24.      I would let my child use hearing aids

25.      I would accept ear surgery for my child

26.      I would like more information

In the Qassim Questionnaire: (Different question)

-               CNS infection can cause HL

-               Neonatal infection can cause HL

-               Maternal Infection can cause HL

-               Radiotherapy can cause hearing loss

-               Chemotherapy can cause HL

-               Consanguineous marriage can cause HL

-               Low birth Weight <1500g can cause HL

-               CHL/SNHL risk related

-               Craniofacial anomalies can cause HL

-               Head trauma can cause HL

-               Recurrent urticaria can cause HL

Appendix 2

Questionnaire in Arabic

استبيان فقدان السمع باللغه العربية

القسم الأول : البيانات الديموغرافية :

１-   العمر :

-        اقل من ٢٥ عامًا

-        ٢٦-٣٥

-        ٣٦-٤٥

-        اكبر من ٤٦ عام

２-   المرحلة التعليمية:

-        الابتدائية

-        المتوسطة

-        الثانوية

-        الجامعية واعلى

３-   المدينة

-        مكة

-        جدة

-        الطائف

-        المدينة المنورة

القسم الثاني :

1.     يمكن أن يولد الأطفال بفقدان السمع

2.     ارتفاع درجة الحرارة يمكن أن يسبب فقدان السمع

3.     يمكن أن تسبب داء الحصبة فقدان السمع

4.     يمكن أن تسبب الام المصابة بداء الحصبة الألمانية فقدان السمع لطفلها

5.     تعاطي المخدرات/الأدوية يمكن أن تسبب فقدان السمع

6.     اليرقان (الصفاري) يمكن أن يسبب فقدان السمع

7.     تأخير البكاء للطفل يمكن أن يسبب فقدان السمع

8.     التاريخ العائلي بفقدان السمع يمكن أن يكون سبب لفقدان السمع

9.     التعرض للضوضاء يمكن أن يسبب فقدان السمع

10.  يمكن للأرواح الشريرة أن تسبب فقدان السمع

11.  اللعنات يمكن أن تسبب فقدان السمع

12.  يمكن أن تؤدي إفرازات الأذن والتهاب الأذن الوسطى إلى فقدان السمع

13.  الانفلونزا المتكررة يمكن أن تسبب فقدان السمع

14.  الرضاعة الطبيعية خلال الأشهر الستة الأولى يمكن أن تقلل/تمنع التهاب الأذن الوسطى

15.  التدخين يمكن أن يسبب التهاب الأذن الوسطى

16.  التحصين (التطعيم) الروتيني في مرحلة الطفولة يمكن أن يقلل من التهاب الأذن الوسطى

17.  يمكن الكشف عن فقدان السمع بعد الولادة مباشرة

18.   يمكن أن تكون مشاكل النطق/اللغة علامة على فقدان السمع

19.  يتوفر علاج لفقدان السمع

20.  يمكن للأطفال الذين يعانون من ضعف السمع الالتحاق بالمدرسة

21.  أرغب في إجراء فحص لطفلي/تي بعد الولادة مباشرة

22.  سأقبل اختبار فحص السمع للانبعاثات السمعيه لطفلي/تي

23.  أرغب في اجراء اختبار السمع لطفلي/تي في المدرسة

24.  سأسمح لطفلي/تي باستخدام أجهزة مساعده لضعف او فقدان

25.  من الممكن ان اسمح بإجراء عملية جراحية لأذن طفلي/تي

26.  ارغب بمعرفة المزيد من المعلومات

من القصيم

27.   التهابات الدماغ من الممكن ان تسبب فقدان للسمع

28.  الالتهابات لدى الأطفال من الممكن ان تسبب فقدان السمع

29.  الالتهابات لدى الأم من الممكن ان تسبب فقدان السمع

30.   العلاج الإشعاعي من الممكن ان يسبب فقدان السمع

31.  العلاج الكيماوي من الممكن ان يسبب فقدان السمع

32.  زواج الأقارب من الممكن ان يسبب فقدان السم

33.  وزن الرضيع اقل ١٥٠٠ جرام من الممكن ان يسبب فقدان السمع

34.   --

35.  تشوهات الوجه من الممكن ان تسبب فقدان السمع

36.  إصابات الرأس من الممكن ان تسبب فقدان السمع

37.   الطفح الجلدي المتكرر من الممكن ان يسبب التهاب الأذن

38.  ما الطريقة المفضلة لأخذ معلومات صحية/ طبية :

-        فيديو تعليمي

-        حملة توعوية

-        الإنترنت

-        تطبيقات التواصل الاجتماعي
